# Supplementary material for: Loneliness and its associated factors among university students during late stage of COVID-19 pandemic: An online cross-sectional study
Source: PLoS One. 2023 Jul 6;18(7):e0287365. doi: 10.1371/journal.pone.0287365 (PMC10325105; doi:10.1371/journal.pone.0287365)
Supplement: Attachment — Submitted filename: ARTICLE REVIEW.docx [file pone.0287365.s002.docx]

**ARTICLE REVIEW**

**TITLE:** Loneliness and its associated factors among University students during late stage of

COVID-19 Pandemic: An online cross-sectional study

**Summary And General Impression**

**Comment**: The authors of the study have collected adequate data information from participants. The paper is generally well written and structured for easy read by researchers. The article was presented in an intelligible fashion and written in standard English. The authors used appropriate reporting format and guidelines in the article.

However, there were few typographical errors and sentence constructions which the authors would have to relook at again to improve understanding and reading. (I have provided some of these below).

a. The authors will need to provide more in-depth analysis of the data to elaborate on the discussion a bit further
b. The authors will need to explain the relevance of the study
c. Further explanations should be provided under the discussion.

**Specific Comment from Reviewer**

|  | Positive comments | Reviewer Comment |  |
| --- | --- | --- | --- |
| Abstract: |  | Conclusion. & Recommendation. L2- rephrase statement, |  |
| Introduction- Authors should rephrase statements | Definition of the loneliness was appropriate.  The general background information is relevant to the study.  Problem statement appropriately stated followed by aims and objectives of the study, and these were well written. | P.2: The higher can be removed…. Higher institutions…  P.2, L3: rephrase statements. It is long.  P. 3, L10 -sentence is long and winding  P. 3-L8- may successfully have reduced… but which…  P.4- could paragraph 4 move up right after paragraph 1? Otherwise, the introduction looks disjointed, following this paragraph. |  |
| Methodology | Research design, setting and period was appropriately stated. | Design- Authors should explain briefly the relevance of the cross-sectional design to the study. Tenses in the sentence should be relooked at, … design was… instead of …has been…  Study population-  L.2- Rephrase sentence, Participation by university students was voluntary, they granted ethical consent….  Eligibility criteria should include age of participants. Age criteria particularly the minimum age was not stated. This is important to ensure that participants gave appropriate consent to participate in the study.  Participants below 18years would need parental consent as well as assent.  Sampling technique- Rephrase second sentence L2).  Data collection and procedure- Authors should mention the full names of tools administered and subsequently use the initials. It would be important that authors provide names of tools used, |  |
| Result | The results describe briefly the demographic data and results from the study. | Socio demographic Characteristics were provided but not details in a tabular for although authors stated table 1. |  |
| Discussion | The discussion was written based on the results. Limitations were stated clearly in the study. | Rephrase statements and some phrases, e.g., P.8 participants who reported sleeping problems instead of self-reported sleeping problem  Please some discussion may need further elaboration. E.g., the odds of loneliness among females being higher would need further explanation. Authors stated … females’ emotion being more sensitive and fragile… as reason however, the study to support that did not find any difference between the male and female gender.  P. 9- Did the study result show students from non-health department developing higher levels of psychological stress?  P.10- harassed female students mostly experience feeling of anger not feeling and anger. 2. Did this study measure feeling of anger? How is this substantiated?  P. 12= this cannot be a paragraph because it is just one statement. Further explanation would help substantiate this finding. |  |
| Conclusion | Generally, the research conclusion was based on the data. |  |  |
| References |  |  |  |
