# Supplementary material for: Loneliness and its associated factors among university students during late stage of COVID-19 pandemic: An online cross-sectional study
Source: PLoS One. 2023 Jul 6;18(7):e0287365. doi: 10.1371/journal.pone.0287365 (PMC10325105; doi:10.1371/journal.pone.0287365)
Supplement: Attachment — Submitted filename: Response to Reviewers.docx [file pone.0287365.s003.docx]

**Response to Reviewers comments**

**Point by point response for editors/reviewers’ comments**

**Manuscript title: Loneliness and its associated factors among University students during late stage of COVID-19 Pandemic: An online cross-sectional study**

**Manuscript ID:** PONE-D-22-01565

**Dear editor/reviewers. Thank you for giving us the chance to revise the manuscript again. Saying this we addressed all the concerns raised by the reviewers and incorporated the authors’ reflection in the revised manuscript.**

**Summary and General Impression**

a. The authors will need to provide more in-depth analysis of the data to elaborate on the discussion a bit further

**Authors’ response**: **Thank you, the authors performed descriptive statistics and logistic regression. We included the result and we improved the discussion in the revised manuscript.**

b. The authors will need to explain the relevance of the study

**Authors’ response**: **we improved the relevant of the study in the revised manuscript.**

c. Further explanations should be provided under the discussion.

**Authors’ response**: **we improved the discussion thoroughly in the revised manuscript.**

**Specific Comment from Reviewer**

**Abstract:**

Conclusion. & Recommendation.

L2- rephrase statement,

**Authors’ response**: **we revised the statement as “**Being female, working in non-health-related fields, having sleeping problems, encountering sexual harassment, perceived stress, and poor social support were significantly associated with loneliness.”

**Introduction-**

Authors should rephrase statements

P.2: The higher can be removed…. Higher institutions…

P.2, L3: rephrase statements. It is long.

P. 3, L10 -sentence is long and winding

P. 3-L8- may successfully have reduced… but which…

P.4- could paragraph 4 move up right after paragraph 1? Otherwise, the introduction looks disjointed, following this paragraph.

**Authors’ response**: **Thank you. We rewrite all above recommendations as suggested (See the revised manuscript).**

**Methodology**

**Design**- Authors should explain briefly the relevance of the cross-sectional design to the study. Tenses in the sentence should be relooked at, … design was… instead of …has been…

**Authors’ response**: **thank you. We amended as “A cross-sectional study design was used among university students in Ethiopia. This study design was chosen because it is relatively fast and inexpensive for population-based surveys to assess the prevalence of loneliness” in the revised manuscript.**

**Study population**- L.2- Rephrase sentence, Participation by university students was voluntary, they granted ethical consent….

**Authors’ response: Thank you. We revised as “University students who were willing to participate and were available online during the study period were enrolled” in the revised manuscript.**

Eligibility criteria should include age of participants. Age criteria particularly the minimum age was not stated. This is important to ensure that participants gave appropriate consent to participate in the study. Participants below 18years would need parental consent as well as assent.

**Authors’ response**: **Thank you. As per the authors know there were no university students below 18 years in Ethiopia. Students start their primary education at the age of 7 and for 12 years up to joining the university. This indicates 18 year is the minimum age for university students. However we accept the reviewers' concern that there may be possibility of this and to make it clear, we included the age as inclusion criteria as suggested “All university students who had used social media (such as Telegram, Facebook, and Imo) and who were above the age of 18 years were included.”**

**Sampling technique-** Rephrase second sentence L2).

**Authors’ response**: **Thank you, we rewrite as “The snowball sampling technique was used in the current study.”**

**Data collection and procedure-** Authors should mention the full names of tools administered and subsequently use the initials. It would be important that authors provide names of tools used,

**Authors’ response**: **Thank you. We included the full names of the tools in the revised manuscript.**

**Result**

Socio demographic Characteristics were provided but not details in a tabular for although authors stated table 1.

**Authors’ response**: **Thank you. Tables were separately submitted. We included tables in the main document (table 1 for sociodemographic characteristics, table 2 loneliness and related information and table 3 for associated factors) in the revised manuscript.**

**Discussion**

Rephrase statements and some phrases, e.g., P.8 participants who reported sleeping problems instead of self-reported sleeping problem

**Authors’ response**: **thank you. We revised the sentences as suggested “Participants who reported sleeping problem was significantly associated with loneliness in the current study”.**

Please some discussion may need further elaboration. E.g., the odds of loneliness among females being higher would need further explanation. Authors stated … females’ emotion being more sensitive and fragile… as reason however, the study to support that did not find any difference between the male and female gender.

**Authors’ response**: **we appreciate the reviewers concern on this statement that was references error. We remove this citation and rewrite and cite the statement gain in the revised manuscript. “This might be due to the fact that females are more sensitive to emotional expressions which is relevant to the antecedents of loneliness (77, 78).”. (**<https://doi.org/10.3389/fnhum.2018.00275> **and doi:10.1093/geront/gnaa082 )**

P. 9- Did the study result show students from non-health department developing higher levels of psychological stress?

**Authors’ response**: **thank you. The result did not assessed the stress among the two groups. But the authors standing point to this explanation was the previous studies that have been cited. We also confirmed that perceived stress was associated with loneliness in the current study as indicate in table 2.**

P.10- harassed female students mostly experience feeling of anger not feeling and anger. 2. Did this study measure feeling of anger? How is this substantiated?

**Authors’ response**: **Thank you, the current study did not assessed anger. We improved this statement as “Harassed female students mostly experience symptom of loneliness and other mental health problems”.**

P. 12= this cannot be a paragraph because it is just one statement. Further explanation would help substantiate this finding.

**Authors’ response**: **Thank you the authors improved this paragraph ay adding the odds ratio and justification. “The current study identified perceived stress as a significant factor for loneliness. Study participants with symptoms of perceived stress had 6.4 time higher risk of loneliness than the counterparts. This is supported by a previous study (****47****). This might be due to negative impact of lifetime stress exposure on mental health (****86****).”.**

**Reviewer #1:**

Loneliness and its associated factors among University students during late stage of COVID-19 Pandemic: An online cross-sectional study titled has fulfilled all the basic and to extent survey made out of loneliness in the pandemic situation.

There are lot of interlocks and chain of reference which found to be disgusting during the reading of the manuscript that could be avoided.

**Authors’ response**: **Thank you, the authors seen the whole document and amend some errors (see the track changes).**

The SPSS survey results could be added as a pictorial representation to figure out the exact result in a better understanding way.

**Authors’ response**: **Thank you, the authors added the SPSS survey results (Table 1 and 2) in the revised manuscript.**

**Reviewer #2:**

The authors did not submit their data with the written article submitted.

**Authors’ response**: **Thank you. The authors added the data as supplementary file.**

The data analysis done was not presented in any format such as tabular format. However, they reported briefly of the findings of the written article, hence the reviewer is not able to review whether the information provided as the data analysis was adequately performed or not.

**Authors’ response**: **Thank you, the authors added the SPSS survey results (Table 1 and 2) in the revised manuscript.**

It would be relevant for the authors of this written article to present a tabular format of the results of the data analysis, or if they can present the result of the analysis as an appendix.

**Authors’ response**: **Thank you, the authors added the SPSS survey results with in the main document (Table 1 and 2) in the revised manuscript and as supplement file.**
